# Characterization of a Single Genomic Locus Encoding the Clustered Protocadherin Receptor Diversity in *Xenopus tropicalis*

**DOI:** 10.1534/g3.116.027995

**Published:** 2016-06-03

**Authors:** Hakki E. Etlioglu, Wei Sun, Zengjin Huang, Wei Chen, Dietmar Schmucker

**Affiliations:** * Neuronal Wiring Laboratory, Vlaams Instituut voor Biotechnologie (VIB), University of Leuven, 3000, Belgium; † Department of Human Genetics, School of Medicine, University of Leuven, 3000 Belgium; ‡ Laboratory for Functional Genomics and Systems Biology, Berlin Institute for Medical Systems Biology, Max-Delbrück-Centrum für Molekulare Medizin, 13125 Berlin, Germany

**Keywords:** *Xenopus tropicalis*, gene cluster, neuronal development, protocadherin receptor diversity

## Abstract

Clustered protocadherins (cPcdhs) constitute the largest subgroup of the cadherin superfamily, and in mammals are grouped into clusters of α-, β-, and γ-types. Tens of tandemly arranged paralogous Pcdh genes of the Pcdh clusters generate a substantial diversity of receptor isoforms. cPcdhs are known to have important roles in neuronal development, and genetic alterations of cPcdhs have been found to be associated with several neurological diseases. Here, we present a first characterization of cPcdhs in *Xenopus tropicalis*. We determined and annotated all cPcdh isoforms, revealing that they are present in a single chromosomal locus. We validated a total of 96 isoforms, which we show are organized in three distinct clusters. The *X. tropicalis* c*Pcdh* locus is composed of one α- and two distinct γ-Pcdh clusters (*pcdh*-γ*1* and *pcdh*-γ*2*). Bioinformatics analyses assisted by genomic BAC clone sequencing showed that the *X. tropicalis* α- and γ-Pcdhs are conserved at the cluster level, but, unlike mammals, *X. tropicalis* does not contain a β-Pcdh cluster. In contrast, the number of γ-Pcdh isoforms has expanded, possibly due to lineage-specific gene duplications. Interestingly, the number of *X. tropicalis* α-Pcdhs is identical between *X. tropicalis* and mouse. Moreover, we find highly conserved as well as novel promoter elements potentially involved in regulating the cluster-specific expression of cPcdh isoforms. This study provides important information for the understanding of the evolutionary history of cPcdh genes and future mechanistic studies. It provides an annotated *X. tropicalis* cPcdh genomic map and a first molecular characterization essential for functional and comparative studies.

The term “protocadherin” (Pcdh) was coined by Suzuki and his colleagues in 1993 (Sano *et al.* 1993), and refers to a large number of cadherin-like cell surface receptors, which are expressed primarily in the nervous system. Clustered protocadherins (cPcdhs) were first discovered in mammals, in which the majority of the Pcdh genes are organized in tandem in a single genomic locus, hence the name “clustered protocadherins” (Kohmura *et al.* 1998; Wu and Maniatis 1999).

Mouse cPcdh genes are organized into α, β, and γ clusters, containing 14, 22, and 22 genes, respectively. The α and γ clusters contain “variable exons” (VEs) as well as “constant exons” (CEs), the latter being located at the distal ends of the clusters. In each mature α- and γ-Pcdh transcript, a VE is spliced to three CEs. The VEs code for the six extracellular cadherin repeats (EC1-6), the transmembrane domain, and a short cytoplasmic tail. The CEs, which are shared by all the isoforms of the same cluster, code for most of the cytoplasmic domain, and are referred to as the “constant domain.” The β cluster is composed of tandem-arrayed single-exon genes lacking constant domains but still retaining a short cytoplasmic domain encoded by each of the VEs.

Based on the phylogenetic analyses of human cPcdhs, two of the 14 α-Pcdhs, and three of the 22 γ-Pcdhs, which form a distinct paraphyletic group, are referred to as the “C-type” cPcdhs. The remaining isoforms are referred to as “non C-type.” For γ-Pcdh , these non C-type cPcdhs are grouped as either “A-type” or “B-type” (Wu and Maniatis 1999). Single-cell PCR studies of cerebellar Purkinje cells revealed that the five C-type isoforms are expressed biallelically and broadly, while the remaining α- and γ-Pcdhs, as well as all of the β-Pcdhs, are expressed monoallelically and in a restricted fashion (Chess 2005; Kaneko *et al.* 2006; Hirano *et al.* 2012). The work of Chen *et al.* (2012) suggested differential roles for the three C-type γ-Pcdhs, as the deletion of these genes resulted in apoptotic loss, or reduced numbers, of some neuronal populations in the spinal cord and in the retina, respectively. In contrast, the deletion of three A-type γ-Pcdhs did not cause any detectable phenotypes (Chen *et al.* 2012).

Studies on the expression of different cPcdhs have discovered several distinct promoter elements, enhancers, as well as intra and intergenic regulatory elements (Wu *et al.* 2001; Golan-Mashiach *et al.* 2012; Ribich *et al.* 2006; Yokota *et al.* 2011; Guo *et al.* 2012; Tan *et al.* 2010). A conserved sequence element (CSE), which is present in most mammalian cPcdh promoters, was shown to constitute a binding site for the CCCTC-binding factor (CTCF) / cohesin complex, which regulates transcription via chromatin modifications and looping (Kehayova *et al.* 2011).

cPcdhs were initially thought to be a vertebrate innovation since they are absent in the invertebrates, while nonclustered Pcdhs, which predate bilaterians, are present in the invertebrate genomes (Hulpiau and van Roy 2009). Recently, Pcdh clusters were identified in octopus; however, vertebrate cPcdhs and clustered octopus Pcdhs do not appear to be direct orthologous genes, but likely result from convergent evolution (Albertin *et al.* 2015).

cPcdh loci are among the most dynamic in vertebrate genomes. Even the slowly evolving genomes of elephant shark and coelacanth have rather dynamic Pcdh loci (Noonan *et al.* 2004a; Yu *et al.* 2008). It was reported that Pcdh clusters experienced repetitive gene duplication, gene loss, gene degeneration, adaptive variation, and gene conversion events (Noonan *et al.* 2004b; Wu 2005). Despite the dynamic nature of Pcdh clusters, a synteny of genes flanking the Pcdh loci is still present, even between mammals and cartilaginous fishes (Yu *et al.* 2008). The elephant shark genome was shown to contain δ-, ε-, μ-, and ν-type cPcdhs, but not α-, β-, or γ-Pcdhs, suggesting that the ancestral vertebrate genome contained a vast repertoire of different Pcdh subclusters, which then formed the modern Pcdh clusters via repetitive lineage-specific gene duplications and losses (Yu *et al.* 2008). The δ-Pcdh subcluster is composed of a single gene. It was shown to be an ancient member of the cPcdhs, being present in diverse vertebrate clades but lost in the mammalian lineage (Yu *et al.* 2008). Zebrafish and fugu, two representative species from the teleost fish lineage, were shown to contain two unlinked Pcdh loci in their genomes (Noonan *et al.* 2004a; Yu *et al.* 2008), due to the whole genome duplication event of teleost fishes. On the other hand, there is only a single cPcdh locus in the ancient genome of coelacanth, a representative of the nonteleost lineage (Noonan *et al.* 2004a).

The complex genomic organization and diverse regulation mechanisms of cPcdhs account for their remarkable diversity in the vertebrate nervous system. cPcdhs were shown to play roles in neuronal self avoidance thanks to their combinatorial expression patterns and strictly homophilic interactions. In this respect, cPcdhs demonstrate striking functional similarity with the *Drosophila* Dscam1 (Zipursky and Sanes 2010; Kise and Schmucker 2013).

The frog, *Xenopus tropicalis*, serves as a model organism, especially for developmental biology, and significant effort has been put into developing molecular tools for genetic analysis analogous to the powerful genetics available for the analysis of mouse. Importantly, the full diploid genome of *X. tropicalis* was recently sequenced, although some gene annotations and gene models still need to be established (Hellsten *et al.* 2010). This applies also to the full *X. tropicalis* cPcdh locus.

In this study, we used cDNA and BAC sequencing, RNA *in situ* expression analysis, as well as bioinformatics analyses, to characterize *X. tropicalis* cPcdhs. We show that there is a single cPcdh locus in the *X. tropicalis* genome containing three clusters, analogous to the mammalian genome organization. In contrast to the mouse locus, however, the *X*. *tropicalis* genome lacks β-Pcdh and instead contains a novel additional γ-cluster (Figure 1). The genomic locus consists of one α, one γ1, and one γ2 cluster. *X. tropicalis* and mouse have the same number of α-Pcdh isoforms (14 in both), while the two *X. tropicalis* γ clusters are significantly larger, with γ1 containing 46 and γ2 containing 36 isoforms. The expansion appears to reflect extensive lineage-specific gene duplication events (58 isoforms in mouse and 96 in *X. tropicalis*). We conducted SMRT sequencing of a BAC clone targeting the γ2 locus (Figure 1), and validated that our sequencing results are in good agreement with sequences released by the *X. tropicalis* sequencing project. We detected low-level gene conversion events exercising on the *X. tropicalis* cPcdhs, at levels comparable to those of mouse cPcdhs.

**Figure 1:**
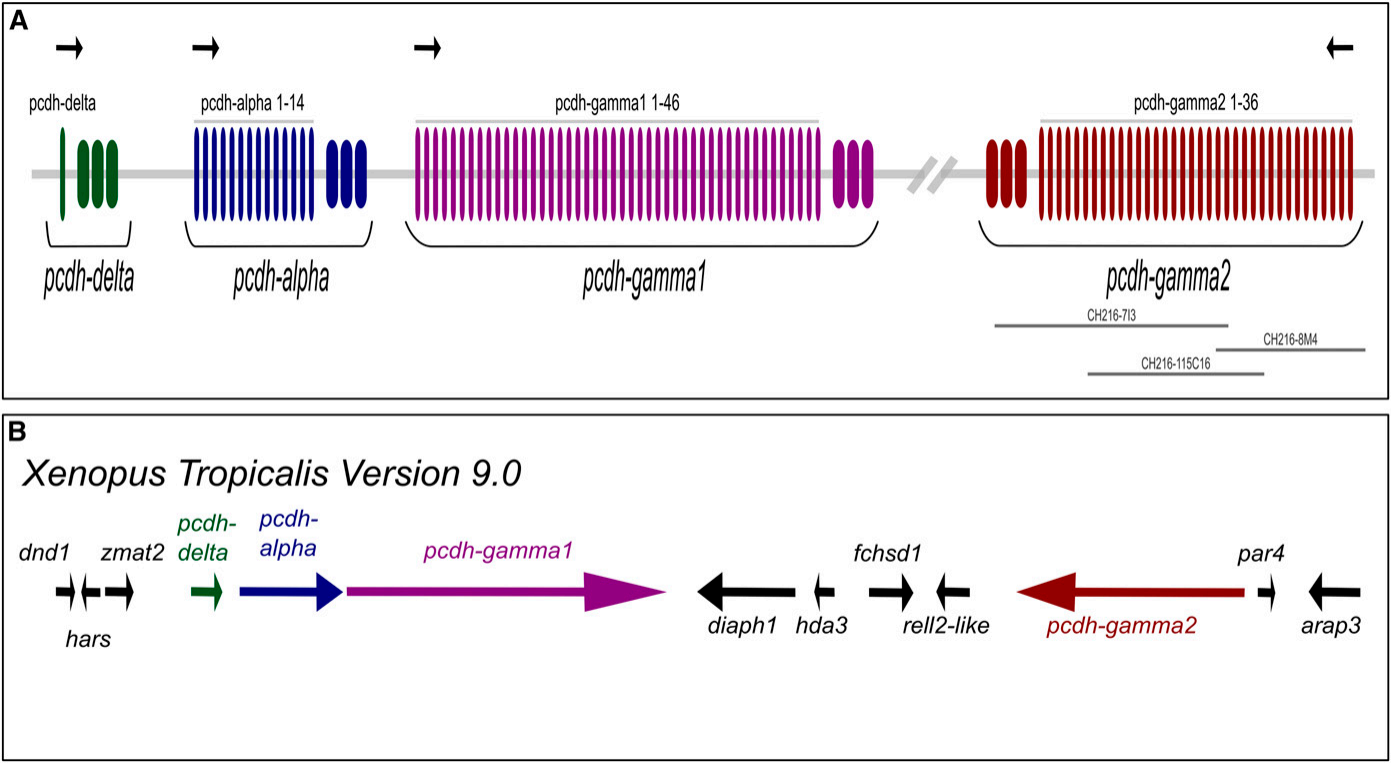
Genomic organization of the *X. tropicalis* cPcdhs. (A) Representative diagram showing *X. tropicalis* cPcdhs gene models. Longer and thinner rectangles denote variable exons, shorter and thicker rectangles denote constant exons. Green: *pcdh*-δ, blue: *pcdh*-α, purple: *pcdh*-γ*1*, red: *pcdh*-γ*2*. Black arrows above the gene models denote the orientation of the clusters. Gray bars below the gene models correspond to the sequenced BAC clones. (B) Genomic map of *X. tropicalis* cPcdhs loci based on genome build 9.0.

## Materials and Methods

### Gene annotation

*X. tropicalis* Genome Build 7 (JGI 7.1/xenTro7) was used for the gene annotation. The annotation was performed manually; gene models were predicted by open reading frame analysis and GENSCAN (Burge and Karlin 1997), and were validated experimentally by the analysis of *X. tropicalis* EST clones from public databases, analysis of cDNA clones from our cluster specific 5′RACE reactions, and reanalysis of publicly available RNA-seq data (Tan *et al.* 2013; Paranjpe *et al.* 2013). Gene-specific 5′RACE primers were designed so as to detect the 3′UTR regions of each cluster in order to be able to detect all of the isoforms of a given cluster. The 5′RACE sequences for the α, γ1 and γ2 clusters are 5′-GGAAGGTGCATCAACAGTAGGAAGAA-3′, 5′-TGCCCTGTTGGTGTCAGCCAATC-3′, and 5′-ACCAATTCGCTTGGGGAATTCTTCTGGGG-3′, respectively.

### BAC sequencing

*X. tropicalis* BAC clone CH216-115C16 was obtained from the CHORI BACPAC Resource Center and purified with the Epicenter BACMAX DNA Purification Kit. Sequencing was performed with Pacific Biosciences SMRT technology using the standard 5-kb template preparation protocol, C2 chemistry, and P4 polymerase, with Magbead loading on SMRT cells, and with a 120-min movie time.

### Phylogenetic analysis

Multiple sequence alignments were produced with CLUSTALW (Sievers *et al.* 2011). The alignments were used to estimate the maximum likelihood (ML) phylogeny with PhyML (Guindon *et al.* 2010), with parameters determined by ModelGenerator (Keane *et al.* 2006).

### Gene conversion

Synonymous substitution rates were estimated with the yn00 program of the PAML package (Yang 2007). Nucleic acid multiple sequence alignments were generated by the RevTrans program (Wernersson and Pedersen 2003) based on amino acid multiple sequence alignments generated with CLUSTALW (Sievers *et al.* 2011).

### Promoter motif discovery

Promoter regions (2 kb upstream of the translational start codon) were scanned with the MEME suite (Bailey *et al.* 2009).

### RNA-seq analysis

Publicly available RNA-seq data were retrieved from the European Nucleotide Archive. Adapter and quality trimming were performed with Trimmomatic (Bolger *et al.* 2014), and reads were mapped to xenTro7 with Tophat2 (Trapnell *et al.* 2009) using the option “–b2-very-sensitive.” Uniquely mapped read-pairs/reads were retrieved with Samtools (Li *et al.* 2009) and were fed to HTSeq for count-based expression analysis (Anders *et al.* 2013). The expression was reported as FPKM or RPKM for paired-end or single-read sequencing, respectively.

### RNA in situ hybridization

Whole mount RNA *in situ* hybridization experiments were performed according to the protocol introduced by Harland (1991). The *pan-gamma* probe targets the constant exons of the γ1 cluster, while the *gamma1*, *gamma2*, and *alpha* probes recognize the 3′UTR regions of the γ1, γ2, and α clusters, respectively. The PCR primer sequences are as follows: *pan-gamma* forward: 5′-CAGGCGCAGCCGAACG-3′, *pan-gamma* reverse: 5′-CAGGCGCAGCCGAACG-3′; *gamma1* forward: 5′-CAGGCGCAGCCGAACGCAGACTGGCGAG-3′, *gamma1* reverse: 5′-GTTCCACTCAGACCCAACTCT-3′, *gamma2* forward: 5′-CAGGCGCAACCTAACGCAGATTGGCGGT-3′, *gamma2* reverse: 5′-TCATTGTTTTGCGTTCTGCGT-3′; *alpha* forward: 5′-CCCAAACATCCTCATCCA-3′; *alpha* reverse: 5′-TCATTTATCGTTGTTGTCAGCT-3′.

### Data availability

The authors state that all data necessary for confirming the conclusions presented in the article are represented fully within the article.

## Results

### X. tropicalis locus containing three variable Pcdh clusters

The gene models of *X. tropicalis* Genome Builds 7 (xenTro7) and 8 (xenTro8) related to proposed cPcdhs had been incomplete at the outset of this analysis. In order to identify genomic regions corresponding to cPcdh genes, we performed TBLASTN searches using mouse α-, β-, and γ-Pcdh, and lizard δ-Pcdh, sequences as queries and the *X. tropicalis* Genome Build 7 (xenTro7) as the subject. We identified an α cluster, two γ clusters, and a single Pcdh-δ gene in a single scaffold (scaffold_3). We did not identify a β cluster, which, in mouse, is situated between α and γ clusters.

The newly identified clusters were annotated manually on Genome Build 7 (xenTro7). First, we created gene models using open reading frame analysis (cutoff: 700 amino acids), and the GENSCAN ab initio gene prediction algorithm (Burge and Karlin 1997). Gene models that fail to code for six cadherin repeats were filtered out.

Experimental support for the gene models came from different sources: 1) we performed RACE experiments in order to generate *X. tropicalis* cPcdh cDNA clones. RACE reactions were performed with total RNA from various developmental stages, and the cluster-specific 5′RACE primers targeted the constant exons of the cluster of interest. 2) We screened the *X. tropicalis* EST databases for cPcdh EST clones. 3) We obtained publicly available *X. tropicalis* RNA-seq studies, and reanalyzed the raw data.

Our annotation efforts resulted in the identification of 14, 46, and 36 genes for the *pcdh*-α, *pcdh*-γ*1* and *pcdh*-γ*2* clusters, respectively. In addition, a single *pcdh*-δ gene was also identified.

While this manuscript was in preparation, *X. tropicalis* Genome Build 9 (xenTro9) was released. All of the aforementioned Pcdh clusters are present on chromosome 3 of this genome build, and Pcdh cluster sequences in the two genome builds demonstrate > 99% sequence identity. Moreover, the *X. tropicalis* cPcdh locus is syntenic with the two *X. laevis* cPcdh loci that are located on the short and long third chromosomes XLA3S and XLA3L, respectively (data not shown). We therefore based our cPcdh genomic map on Genome Build 9 (Figure 1).

The clusters are flanked by non-Pcdh genes, suggesting that cPcdh clusters, as annotated in Figure 1, are likely to be complete Pcdh clusters. The *pcdh*-γ*1* cluster can be regarded as syntenic with the mouse γ-Pcdh cluster, while the *pcdh*-γ*2* cluster potentially originated from a duplication of the *pcdh*-γ*1* cluster, and this duplication does not include any flanking genes.

### The assembly of X. tropicalis Pcdh clusters is of high quality

Sequence analysis of the paralogous genes of the *X. tropicalis* Pcdh clusters revealed high similarity between some of the paralogous genes. We defined similarity as percent sequence identity of isoform pairs over their second and third extracellular domains (EC2–EC3) at the amino acid level. EC2–EC3 was chosen as these two ecto-domains were shown to be responsible for providing the specificity of the strictly homophilic interactions of α-, β-, and γ-Pcdhs (Schreiner and Weiner 2010; Thu *et al.* 2014; Rubinstein *et al.* 2015; Nicoludis *et al.* 2015), and hence are expected to be diverse in sequence. Comparison of EC2–EC3 regions of *X. tropicalis* cPcdh isoforms at the amino acid level showed that a large number of *X. tropicalis* cPcdh isoforms demonstrate high sequence identity to at least one other isoform (Supplemental Material, Figure S1). In contrast, the number of “highly similar” mouse cPcdh isoforms is relatively low (Figure S1).

The *X. tropicalis* genome was sequenced with a whole genome shotgun sequencing approach, which sometimes impairs the sequence assembly efforts, especially over complex genomic regions containing repeats, such as the complex cPcdh loci. In order to validate the presence of the highly similar *X. tropicalis* cPcdh as well as to rule out a probable sequence assembly error, which was the case for fugu cPcdh loci in the fugu draft genome assembly (Yu *et al.* 2008), we resequenced part of the *X. tropicalis* cPcdh locus. To this end, we identified a BAC covering *pcdh*-γ*2* isoforms 9 to 31 (170,363 kb), which included nine highly similar isoforms (*pcdh*-γ*2 16–18*, three isoforms; *pcdh*-γ*2 20–25*, six isoforms). The BAC was sequenced with high coverage (average > 1149) using single-molecule real time (SMRT) sequencing technology, which was shown to be capable of resolving complex genomic loci (Huddleston *et al.* 2014). The reads were assembled with HGAP (Chin *et al.* 2013), and, after vector trimming and filtering out lower-coverage regions, the newly assembled contig demonstrated 99.83% sequence identity with the corresponding region of Genome Build 7 (xenTro7). Moreover, all of the nine highly similar isoforms could be identified in the newly assembled contig. This result confirmed the presence of highly similar paralogous cPcdh genes in the *X. tropicalis* genome and provides evidence that the sequence assembly of the current genome build of *X. tropicalis* is of high quality for the cPcdh locus.

### Phylogenetic analysis of X. tropicalis cPcdhs

**Figure 2:**
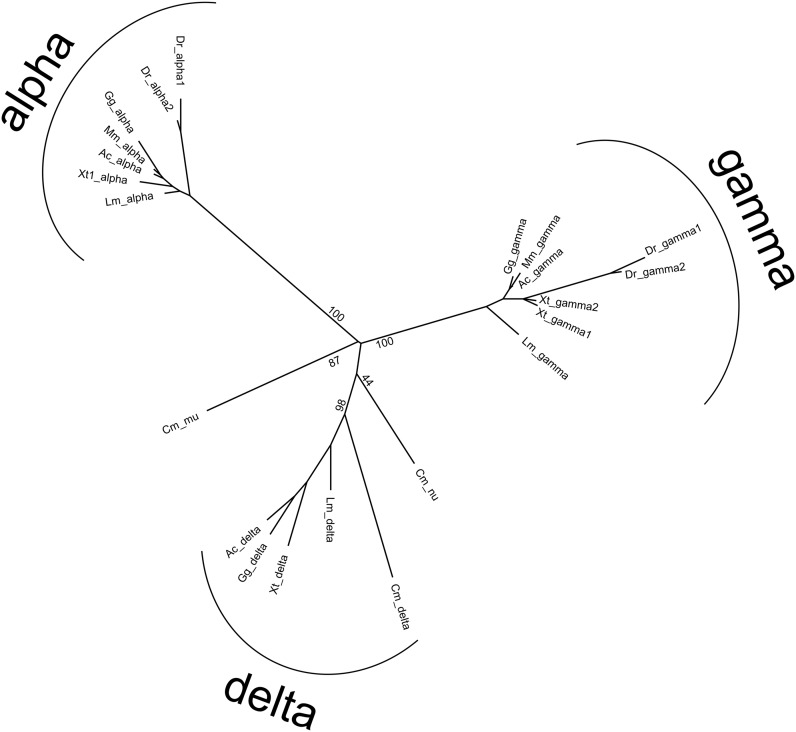
Phylogenetic analysis of cPcdhs at the cluster level. The tree was generated using ML and is based on the multiple sequence alignment of the constant domains of different Pcdh clusters. % Bootstrap values of major nodes are shown. Ac, anole; Cm, elephant shark; Dr, zebrafish; Gg, chicken; Lm, coelacanth; Mm, mouse; Xt, *X. tropicalis*.

In order to investigate the evolutionary relationships of *X. tropicalis* cPcdhs at the cluster level, we constructed a phylogenetic tree using multiple sequence alignments of the constant domains of the cPcdhs of mouse, chicken, lizard, zebrafish, coelacanth, and elephant shark, which represent mammals, birds, reptiles, teleost fish, and lobe-finned fish, respectively, along with the constant domains of *X. tropicalis* cPcdhs (Figure 2). The results confirmed that *X. tropicalis* clusters are direct orthologs of mouse α- and γ-, as well as fish δ-Pcdhs. The phylogenetic tree shows that α- and γ-Pcdhs have a slower evolutionary rate than δ-Pcdhs, suggesting potentially more conserved functions over the course of vertebrate evolution.

Zebrafish and *X. tropicalis* γ cluster isoforms do not form monophyletic groups, which suggests that the duplication events that led to multiple γ clusters in these two species were independent.

### Phylogenetic relationships of mouse and X. tropicalis cPcdhs

**Figure 3:**
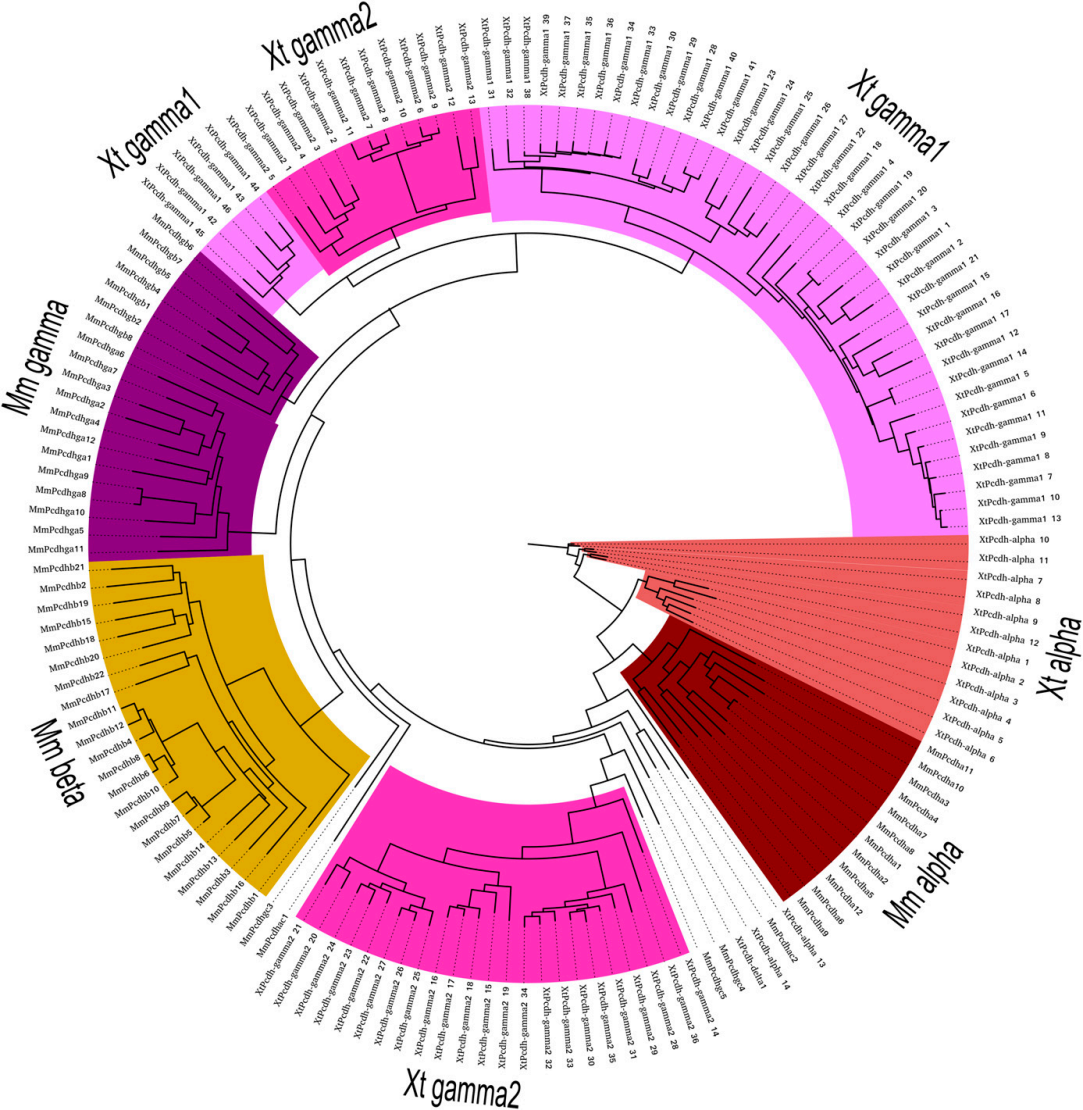
Phylogenetic relationships of mouse and *X. tropicalis* cPcdhs. The tree was generated with ML using the multiple sequence alignments of EC2–EC3 of mouse and *X. tropicalis* cPcdh isoforms.

The analysis of the ORFs encoded by the different *X. tropicalis* Pcdh clusters (utilizing genomic as well as long-read cDNA sequences), revealed that the overall protein domain architecture of *X. tropicalis* is identical to the domain architecture of mammalian α- and γ-Pcdh proteins. All cPcdhs of *X. tropicalis* (as mapped and indicated in Figure 1) contain six extracellular cadherin-repeat domains (EC1–EC6). Assessing the conservation pattern of individual domains based on mouse and *X. tropicalis* amino acid sequences reveals, on average, a higher conservation of domains EC1 and EC5 compared to domains EC2–EC4. This conservation pattern is similar between mouse and *X. tropicalis* (Table 1, Figure 3, Figure S4, and Figure S5). In addition, a direct *trans*-species comparison of a few representative mouse and *X. tropicalis* isoforms (Figure S3, Figure S4, Figure S5, and File S1 quantification in Table 1) indicate good conservation of domains overall, and supports the notion that the *X. tropicalis* cPcdh proteins most likely retain their canonical Pcdh properties (*e.g.*, homophilic binding properties).

**Table 1:** Percent amino acid sequence identity values of the extracellular and cytoplasmic domains of mouse and *X. tropicalis* cPcdhs

Domain	A Average of All Mouse α Isoforms	B Average of All Xt α Isoforms	C Mouse Pcdha9 *vs.* Xt pcdh-α1	D Average of All Mouse γ Isoforms	E Average of All Xt γ1 Isoforms	F Average of All Xt γ2 Isoforms	G Mouse Pcdhga3 *vs.* Xt pcdh-γ1 3	H Mouse Pcdhga3 *vs.* Xt pcdh-γ2 35
EC1	95.85	70.79	57.83	55.80	71.01	56.37	19.85	15.44
EC2	69.29	68.06	52.48	57.63	68.65	58.63	23.91	26.21
EC3	58.52	59.30	45.00	50.48	64.69	51.81	17.65	14.60
EC4	85.49	61.60	49.48	59.32	71.25	57.08	17.16	16.42
EC5	98.01	73.40	46.08	62.76	68.97	59.17	23.74	19.42
EC6	84.31	56.22	38.89	81.62	79.67	62.59	19.35	24.19
CD	100.00	100.00	68.39	100.00	100.00	100.00	75.00	73.60

Columns A, B, D, E, and F show average percent sequence identity values of mouse α, Xt α, mouse γ, Xt γ1, and Xt γ2 clusters, respectively. Columns C, G, and H show percent sequence identity values of mouse Pcdha9–Xt pcdh-α1, mouse Pcdhga3–Xt pcdh-γ1 3, and mouse Pcdhga3–Xt pcdh-γ2 35 pairs, respectively. EC, extracellular domain; CD, constant domain.

On average, the similarity of EC domains between mouse and *X. tropicalis* is moderately lower for the γ2-Pdh cluster as compared to the α-Pcdh and γ1-Pcdh clusters, suggesting a higher degree of divergence for γ2-Pcdh (Table 1). Importantly, the high similarity of the constant cytoplasmic domains (68–75%; Table 1) suggests a high degree of conservation in cytoplasmic effector interactions and biological functions of mouse and *X. tropicalis* cPcdh.

In order to characterize the individual paralogous genes/isoforms of the *X. tropicalis* Pcdh clusters, we generated a phylogenetic tree using the multiple sequence alignments of the amino acid sequences of the second and third extracellular Cadherin repeat region (*i.e.*, EC2 plus EC3 domains “EC2-3”) of each of the mouse and *X. tropicalis* cPcdh isoforms. We focused our comparison on EC2-3 as these two domains were shown to account for the specificity of the homophilic *trans*-interactions of cPcdh (Schreiner and Weiner 2010; Thu *et al.* 2014; Rubinstein *et al.* 2015; Nicoludis *et al.* 2015), and are, therefore, thought to contain key sequences responsible for the functional specificity of a given isoform. Moreover, EC2-3 appears to be prone to a lower frequency of gene conversion events compared to the C-terminal ectodomains EC4-6, and hence carry most of the phylogenetic signal (Noonan *et al.* 2004b).

The phylogenetic tree suggests that no clear orthology relation between the individual genes of mouse and *X. tropicalis* Pcdh clusters is present, and that mouse and *X. tropicalis* Pcdh genes are orthologous only as paralog subgroups. One exception to that is the mouse Pcdhac2 and *X. tropicalis pcdh*-α *14* pair, for which a clear one-to-one orthology can be inferred from the phylogenetic tree. Both isoforms are encoded by the most distal variable exons of their respective α clusters. Mouse *Pcdhac2* was reported to be a C-type cPcdh isoform, which exhibits significant differences in expression compared to A/B-type cPcdhs. Therefore, the relatively shorter evolutionary distance between mouse *Pcdhac2* and *X. tropicalis pcdh*-α *14* further supports the notion of important and specialized functions of these C-type isoforms. In addition, *X. tropicalis pcdh*-α *13* is evolutionarily closer to the C-type Pcdh isoforms than to the *pcdh*-α *1–12* isoforms of its parent α cluster. This analysis suggests that *X. tropicalis pcdh*-α *13* and *pcdh*-α *14* might belong to the distinct C-type cPcdhs, analogous to their mammalian counterparts.

None of the *X. tropicalis* γ-Pcdh showed a clear one-to-one orthology with mouse γ-Pcdh (Figure 3). However, 23 of the 36 isoforms of the *pcdh*-γ*2* cluster (*pcdh*-γ*2 14–36*) are evolutionarily closer to the C-type α- and γ-Pcdh of mouse, and to *X. tropicalis pcdh*-α *13*, *pcdh*-α *14* than their sister isoforms of the *pcdh*-γ*2* cluster or the γ-Pcdhs of the *X. tropicalis pcdh*-γ*1* cluster. This *pcdh*-γ*2 14–36* subgroup constitutes the distal part of the *pcdh*-γ*2* cluster and might be involved in distinct functions comparable to that of the mouse C-type cPcdh isoforms. This also suggests the possibility that there is a significant expansion of the C-type cPcdhs in *X. tropicalis*. In addition to the phylogeny inferred from EC2-3 domain comparisons, we generated a phylogenetic tree with full length sequences of mouse and *X. tropicalis* cPcdh (Figure S2). The topologies of both phylogenetic trees are similar. This observation is consistent with the relatively lower divergence of *X. tropicalis* EC2-3 regions (Table 1), as well as with the lower levels of gene conversion events in *X. tropicalis*.

### X. tropicalis cPcdhs are prone to low level gene conversion events

It was shown that cPcdhs of mouse, human, rat, zebrafish, fugu, coelacanth, elephant shark, and lizard are subject to gene conversion although to different extents (Jiang *et al.* 2009; Noonan *et al.* 2004a, 2004b; Yu *et al.* 2008, 2007). To determine the frequency of gene conversion events acting on the *X. tropicalis* cPcdhs, we calculated the average of “total number of synonymous substitutions per codon” (dS) for each of the major monophyletic groups observed in the above-mentioned phylogenetic analyses (Figure 3). dS is used as an indication of gene conversion since synonymous sites are not affected by functional constraints at the protein level; therefore, a higher number of synonymous substitutions reflects a lower rate of gene conversion events. Table 2 lists the average dS values for each of the six ectodomains of the *X. tropicalis* and mouse cPcdhs as well as the average dS values for a selected group of zebrafish cPcdhs (*Pcdh2ab1–12*), which were shown to be subject to a high frequency of gene conversion events (Noonan *et al.* 2004b). The last column of Table 2 lists the ratio of the highest and the lowest dS values, and is reflective of overall gene conversion events exercising over the entire length of EC1-6. In general, the highest dS values listed in Table 2 were observed in EC2 or EC3 domains, while the lowest dS values were observed in EC5 or EC6 domains (Table 2), which is concordant with previous findings. The ratios of the highest dS and lowest dS for each of the monophyletic groups are comparable for *X. tropicalis* and mouse, but significantly lower than zebrafish. These findings show that *X. tropicalis* cPcdhs experienced similar levels of gene conversion events as mouse cPcdhs, but significantly lower levels of gene conversion events compared to zebrafish cPcdhs.

**Table 2:** Average synonymous substitutions per codon (dS)

	dS_EC1_	dS_EC2_	dS_EC3_	dS_EC4_	dS_EC5_	dS_EC6_	dS_highest_/dS_lowest_
Xt pcdh-α 1-14	1.15	2.84	2.11	1.84	1.27	1.35	2.47
Xt pcdh-γ2 20-26	0.28	0.33	0.68	0.43	0.44	0.33	2.46
Xt pcdh-γ2 28-35	0.46	0.45	0.79	0.47	0.82	0.15	5.35
Xt pcdh-γ2 1-13	1.24	2.55	2.62	1.44	1.13	1.72	2.32
Xt pcdh-γ1 42-46	0.26	1.06	0.63	0.50	1.02	0.53	4.06
Xt pcdh-γ1 1-41	1.67	1.76	1.83	1.64	1.23	0.89	2.04
Mm Pcdha1-12	0.31	2.08	2.15	1.71	0.27	1.35	7.86
Mm Pcdhb1-22	1.08	1.66	1.43	1.60	0.79	1.36	2.10
Mm Pcdhga1-12	2.16	2.29	2.45	2.61	2.34	1.88	1.39
Mm Pcdhgb1-8	1.38	1.90	2.21	1.54	0.60	1.50	3.70
Dr Pcdh2ab1-12	1.67	1.13	2.91	1.13	0.12	0.01	231.14

### Core promoter elements of the X. tropicalis cPcdhs

Promoters of cPcdhs of various species including human, mouse, and zebrafish carry a core element referred to as the CSE. In mouse, the CSE is positioned ∼200 bp upstream of the translational start codon (Wu *et al.* 2001; Noonan *et al.* 2004b), and is composed of a “CGCT box,” which is surrounded by additional conserved cluster-specific sequences. However, zebrafish α-Pcdhs have divergent CSEs that lack the CGCT box (Noonan *et al.* 2004b).

**Figure 4:**
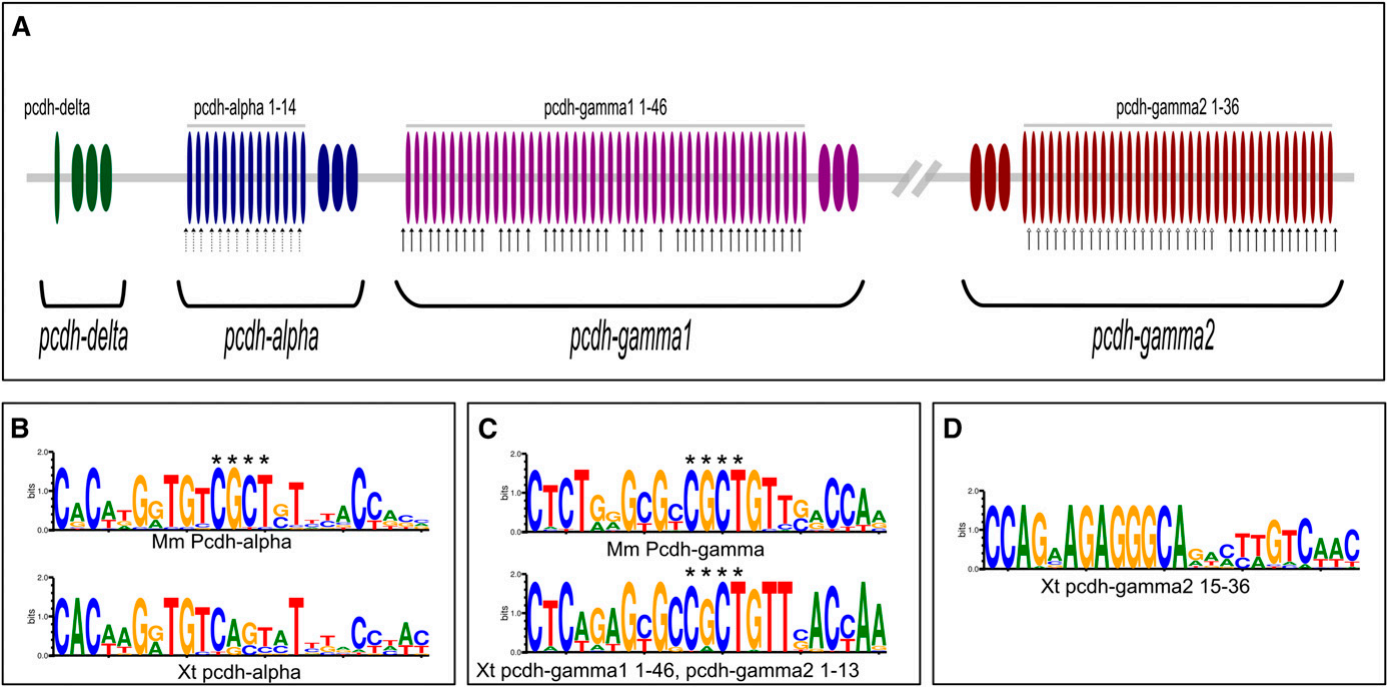
(A) Schematic representation of the conserved sequence motifs of *X. tropicalis* cPcdh promoters. Dotted arrows, filled arrows, and open arrows represent the genomic position of conserved motifs of (left–right) pcdh-α, pcdh-γ1 and pcdh-γ2. (B) CSE of mouse α-Pcdhs and *X. tropicalis* pcdh-α. (C) CSE of mouse γ-Pcdhs and *X. tropicalis* pcdh-γ1 and pcdh-γ2 1–13. (D) The newly identified sequence motif present in the promoters of pcdh-γ2 15–36. The asterisks indicate the “CGCT box.”

Our analysis revealed that many of the *X. tropicalis* cPcdh transcription units also contain the CSE signature sequences found in mammals in their promoters (Figure 4). In addition, we identified a novel distinct conserved promoter sequence motif, which may constitute an additional CSE-like element; however, this remains to be validated experimentally.

We scanned the promoters (2 kb upstream of the translational start codon) of *X. tropicalis* cPcdhs, and showed that the CSE is present in most *X. tropicalis* cPcdh promoters, and, similar to the mammalian CSE elements, they are located some 200 bp upstream of the translational start codon (Figure 4A).

All of the 14 α-Pcdhs of *X. tropicalis* carry the CSE motif; however, the CGCT box, which is conserved in mammalian α-Pcdh promoters, is not present in *X. tropicalis* α-Pcdh promoters (Figure 4B). In contrast, the *X. tropicalis pcdh*-γ*1 1-46* and *pcdh*-γ*2 1-13* (except for *pcdh*-γ*1 isoforms* 11, 16, 25, and 29) carry the CSE motif, in which the CGCT box is conserved, although to a lesser extent than in mammals (Figure 4C).

Isoforms that lack the CSE motif in their promoters (*pcdh*-γ*2 15–36*), carry a 13 bp conserved sequence ∼200 bp upstream of their translational start codon (Figure 4D). This motif has previously not been reported in any other the species, and is not listed in the databases of transcription factor binding sites. Interestingly, this motif is present in a contiguous group of isoforms of the γ2 cluster (*pcdh*-γ*2 15–36*), which form a monophyletic group with mouse C-type cPcdh isoforms *Pcdhgc4* and *Pcdhgc5* (Figure 3).

### Expression patterns of X. tropicalis cPcdhs

In order to further characterize *X. tropicalis* cPcdhs, we investigated their temporal and spatial expression patterns by reanalyzing publicly available RNA-seq data and by RNA *in situ* hybridization.

Tan *et al.* (2013) studied the *X. tropicalis* transcriptome in 23 distinct developmental stages (between the two-cell stage and stage 45) by paired-end RNA sequencing (RNA-seq). For their analyses, they used Genome Build 4 (xenTro3), and gene models associated with this genome build.

This study provided important information about the *X. tropicalis* transcriptome in general; however, due to lack of complete gene models of cPcdhs in Genome Build 4, their expression could not be retrieved directly. Therefore we reanalyzed the raw sequencing data using Genome Build 7 (xenTro7), and our newly created cPcdh gene models. We determined “cluster-level” expression by using or read-pairs that map to the constant exons that are shared by all the genes of a given cluster. The expression levels were reported as “fragments per kilobase of exon per million fragments mapped” (FPKM), which is analogous to the “reads per kilobase of exon per million reads mapped” (RPKM) used in the original study (Tan *et al.* 2013). RPKM (and FPKM) allows transcript level comparisons within, as well as between, samples (Dillies *et al.* 2013).

**Figure 5:**
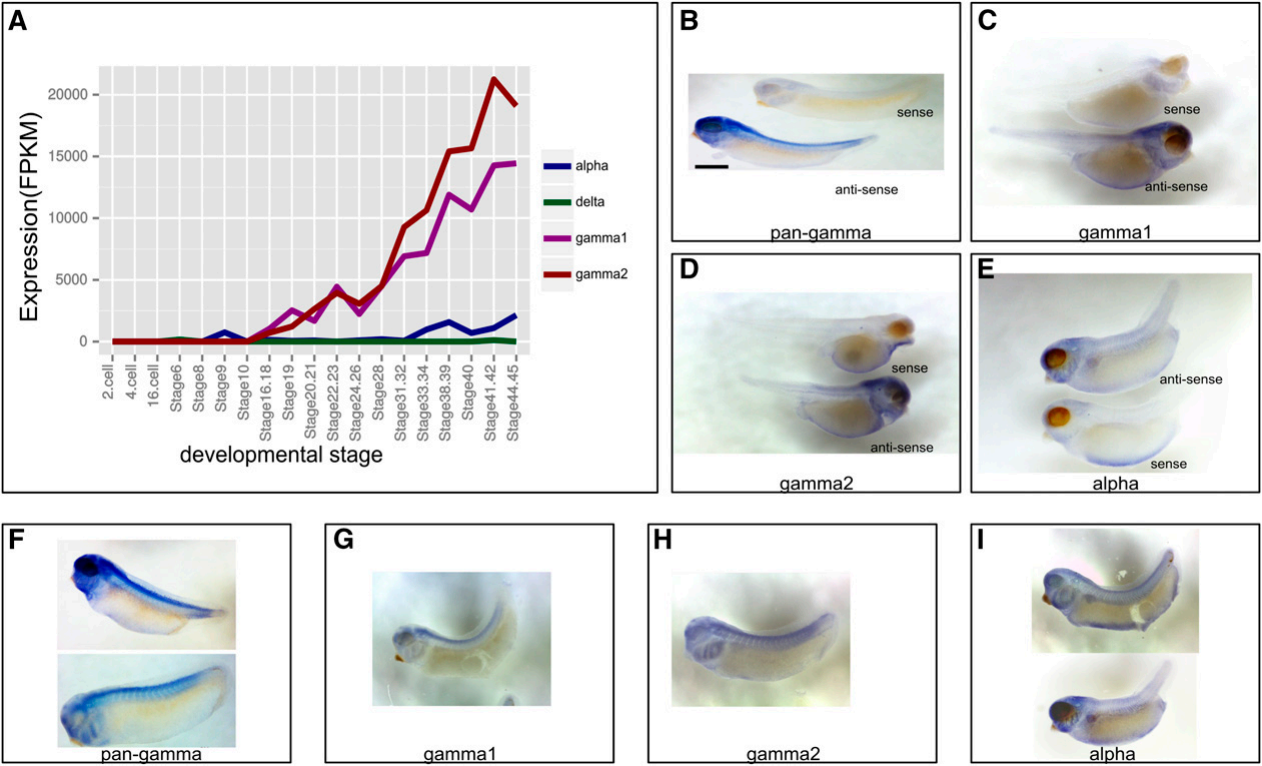
Expression of *X. tropicalis* cPcdhs. (A) *X. tropicalis* cPcdh expression over 19 different developmental stages in FPKM units (reanalyzed from Tan *et al.* 2013). (B–E) *X. tropicalis* cPcdh RNA *in situ* hybridization sense and antisense probes. (F–I) Spatial expression patterns of *X. tropicalis* cPcdhs as detected by RNA *in situ* hybridization. (B–I) mRNA *in situ* hybridization results of the *X. tropicalis* cPcdhs. The *pan-gamma* riboprobe targets the constant exons of *pcdh*-γ*1*, and most likely recognizes the *pcdh*-γ*2* cluster constant exons as well because of high sequence similarity of the constant exons of the two γ clusters (73.89% sequence identity). The *gamma1* and *gamma2* riboprobes recognize the 3′UTR regions of *pcdh*-γ*1* and *pcdh*-γ*2* clusters, respectively, and the *alpha* riboprobe recognizes the constant exons of the *pcdh*-α cluster. (B–E) Sense and antisense probes. (F–I) Additional images from the antisense probes.

The α- and δ-Pcdhs are expressed in low-levels up to stage 45, however α-Pcdh expression levels start to increase at stages 31–32 (Figure 5A). Conversely, the two γ-Pcdhs start to be expressed after stage 12, which marks the end of the gastrula stage and the beginning of the neurula stage. RNA *in situ* hybridization experiments (Figure 5, B–I) show that *X. tropicalis* cPcdh genes from all three clusters are expressed throughout the nervous system. α- as well as γ-Pcdh mRNA is already present in the early neuroectoderm region of embryos (data not shown), and their expression increases through subsequent stages of neural development (Figure 5) and persists into metamorphic stages (data not shown). This includes expression in the eye anlagen, retina, otic vesicle, olfactory bulb, all brain regions, spinal cord, and, at least in part, in neural crest lineages (*e.g.*, expression in pharyngeal arches) (Figure 5). Based on whole-mount *in situ* staining, it is clear that α- and γ-Pcdh are broadly expressed in the nervous system and exhibit substantial overlap in expression. However, whether α- and γ-Pcdh are indeed also coexpressed at the single cell level, and in most or all neurons, awaits further analysis.

## Discussion

In our efforts to characterize the *X. tropicalis* cPcdh receptors, we identified a single chromosomal locus containing three Pcdh clusters that we designated *pcdh*-α, *pcdh*-γ*1* and *pcdh*-γ*2*. The *pcdh*-α cluster contains 14, the *pcdh*-γ*1* cluster contains 46, and the *pcdh*-γ*2* cluster 36 variable exons encoding for a total of 96 Pcdh isoforms. The organization of three tandem arrays of Pcdh isoforms at a single chromosomal location is analogous to the gene organization in mammals and coelacanth (Wu *et al.* 2001; Noonan *et al.* 2004a). While *X. laevis* and zebrafish have multiple cPcdh loci due to whole-genome duplications, we found no evidence of additional Pcdh loci in *X. tropicalis*, which should facilitate future genetic analysis of cPcdh function in this vertebrate model organism.

In contrast to the mammalian cPcdh locus, the *X. tropicalis* genome lacks Pcdh-β receptors, yet contains a second distinct *pcdh*-γ cluster. It seems most likely that the two γ clusters are derived from lineage-specific gene duplications in *Xenopus*; however, a gene loss in other organisms can currently not be excluded based on available sequence information. Pcdh-β receptors are present in mammals (22 isoforms in mouse and 15 in humans), but also in coelacanth, lizard, and chicken genomes. However, little is known about their potential functional specialization and significance, as genetic studies in mice have focused primarily on characterizing the importance of α or γ receptors. Nevertheless, the striking increase in pcdh-γ isoforms in *X. tropicalis* might suggest some degree of functional interchangeability between pcdh-γ and pcdh-β receptors.

It has recently been proposed that cPcdhs emerged in jawed vertebrates, in which a common ancestor comprised numerous different cPcdh types (α, β, γ, δ, ε, μ, ν, *etc*.) (Yu *et al.* 2008). Of those, the α-, β-, γ- and δ-types were retained in bony vertebrates. The fact that coelacanths (nonteleost fish) have a β-Pcdh cluster, while representatives of teleost fishes (zebrafish and fugu) were found to lack β-Pcdhs, suggests that β-Pcdhs were lost in the teleost fish lineage but not all lower vertebrates. In summary, the loss of a β-Pcdh cluster in the *Xenopus* lineage on top of an expanded γ-Pcdh diversification in *X. tropicalis* provides further evidence for an exceptionally dynamic evolution of Pcdh gene clusters.

In a broader context, the high degree of variability of the numbers of clusters, subtypes and isoforms of cPcdh receptors across species might support the notion that evolutionary selection primarily drives the generation of receptor diversity rather than the conservation of unique structural specificities of distinct cPcdh genes. This notion would be consistent with the hypothesis that vertebrate cPcdh receptors—analogous to the hypervariable Dscam1 receptor in flies—function primarily in providing a highly diverse pool of cell adhesion receptors with homophilic binding preferences. Studies in flies have shown that a significant amount of Dscam1 diversity is essential for providing unique surface labels for neurons to support a system of “neuronal self-recognition.” Furthermore, genetic manipulations have shown that, as long as the overall repertoire of receptor isoforms encoded by the Dscam1 gene is above a certain threshold (*e.g.*, a few thousand in flies), such that it can ensure that interacting neurons express multiple nonidentical sets of isoforms, the number and binding specificities of isoforms can vary substantially (Hattori *et al.* 2009). Not surprisingly, the overall number of Dscam1 isoforms in different insect species can vary substantially (Brites and Pasquier 2015).

Our phylogenetic comparisons of cPcdh sequences indicate similar levels of gene conversion among cPcdh isoforms in *X. tropicalis* and mouse, yet significantly lower levels of gene conversions than zebrafish. More importantly, we found striking differences of gene conversion rates when different extracellular cPcdh domains were compared. Specifically, the EC2 and EC3 domains appear not to be subject of gene conversion, in contrast to other extracellular domains of cPcdhs. This is particularly interesting as recent *in vitro* and structural studies suggested that the EC2 and EC3 domains provide essential protein interfaces determining homophilic binding specificity in cPcdh dimers. These data, therefore, corroborate a positive selection of sequences in EC2/EC3 domains that are optimized for unique homophilic binding specificities.

In our comparative analysis, we also found a remarkably high degree of conservation of noncoding sequence motifs serving regulatory functions of cPcdh expression. In fact, many of the *X. tropicalis* cPcdhs variable exon units contain CSE signature sequences characteristic of related promoters in mammalian cPcdhs. This strongly suggests that transcriptional control factors and mechanisms (including chromatin regulation) are highly conserved between frog and mammalian cPcdhs. It is intriguing to note, however, that a distinct set of 22 tandemly arrayed isoforms (*pcdh*-γ*2 15–36*) lacks a CSE motif in their promoters, and yet all contain a similarly positioned currently uncharacterized sequence motif. This suggests the presence of a novel regulatory factor, which may support or drive *X. tropicalis* specific specializations in cPcdh expression and function.

## Supplementary Material

Supplemental Material

## References

[bib1] Albertin C. B. Simakov O. Mitros T. Wang Z. Y. Pungor J. R., 2015 The octopus genome and the evolution of cephalopod neural and morphological novelties. Nature 524(7564): 220–224. 26268193 10.1038/nature14668 PMC4795812

[bib2] Anders S. McCarthy D. J. Chen Y. Okoniewski M. Smyth G. K., 2013 Count-based differential expression analysis of RNA sequencing data using R and Bioconductor. Nat. Protoc. 8(9): 1765–1786. 23975260 10.1038/nprot.2013.099

[bib3] Bailey T. L. Boden M. Buske F. A. Frith M. Grant C. E., 2009 MEME suite: tools for motif discovery and searching. Nucleic Acids Res. 37(Suppl. 2): 202–208. 10.1093/nar/gkp335 PMC2703892 19458158

[bib4] Bolger A. M. Lohse M. Usadel B., 2014 Trimmomatic: a flexible trimmer for Illumina sequence data. Bioinformatics 30(15): 2114–2120. 24695404 10.1093/bioinformatics/btu170 PMC4103590

[bib5] Brites D. Du Pasquier L., 2015 Somatic and germline diversification of a putative immunoreceptor within one phylum: Dscam in arthropods, pp. 131–158 in *Pathogen–Host Interactions: Antigenic Variation v. Somatic Adaptations*, edited by Hsu E. Du Pasquier L. Springer International Publishing, Cham, Switzerland. 10.1007/978-3-319-20819-0_6 26537380

[bib6] Burge C. Karlin S., 1997 Prediction of complete gene structures in human genomic DNA. J. Mol. Biol. 268(1): 78–94. 9149143 10.1006/jmbi.1997.0951

[bib36] Chen W. V. Alvarez F. J. Lefebvre J. L. Friedman B. Nwakeze C., 2012 Functional significance of isoform diversification in the protocadherin gamma gene cluster. Neuron 75(3): 402–409. 22884324 10.1016/j.neuron.2012.06.039 PMC3426296

[bib7] Chess A., 2005 Monoallelic expression of protocadherin genes. Nat. Genet. 37(2): 120–121. 15678142 10.1038/ng0205-120

[bib8] Chin C.-S. Alexander D. H. Marks P. Klammer A. A. Drake J., 2013 Nonhybrid, finished microbial genome assemblies from long-read SMRT sequencing data. Nat. Methods 10(6): 563–569. 23644548 10.1038/nmeth.2474

[bib9] Dillies M. A. Rau A. Aubert J. Hennequet-Antier C. Jeanmougin M., 2013 A comprehensive evaluation of normalization methods for Illumina high-throughput RNA sequencing data analysis. Brief. Bioinform. 14(6): 671–683. 22988256 10.1093/bib/bbs046

[bib10] Golan-Mashiach M. Grunspan M. Emmanuel R. Gibbs-Bar L. Dikstein R., 2012 Identification of CTCF as a master regulator of the clustered protocadherin genes. Nucleic Acids Res. 40(8): 3378–3391. 22210889 10.1093/nar/gkr1260 PMC3333863

[bib11] Guindon S. Dufayard J.-F. Lefort V. Anisimova M., 2010 New algorithms and methods to estimate maximum-likelihoods phylogenies: assessing the performance of PhyML 3.0. Syst. Biol. 59(3): 307–321. 20525638 10.1093/sysbio/syq010

[bib12] Guo Y. Monahan K. Wu H. Gertz J. Varley K. E., 2012 CTCF/cohesin-mediated DNA looping is required for protocadherin α promoter choice. Proc. Natl. Acad. Sci. USA 109(51): 21081–21086. 23204437 10.1073/pnas.1219280110 PMC3529044

[bib13] Harland R. M., 1991 In situ hybridization: an improved whole-mount method for *Xenopus* embryos. Methods Cell Biol. 36: 685–695. 1811161 10.1016/s0091-679x(08)60307-6

[bib38] Hattori D. Chen Y. Matthews B. J. Salwinski L. Sabatti C., 2009 Robust discrimination between self and non-self neurites requires thousands of Dscam1 isoforms. Nature 461(7264): 644–648. 19794492 10.1038/nature08431 PMC2836808

[bib14] Hellsten U. Harland R. M. Gilchrist M. J. Hendrix D. Jurka J., 2010 The genome of the Western clawed frog *Xenopus tropicalis*. Science 328(5978): 633–636. 20431018 10.1126/science.1183670 PMC2994648

[bib15] Hirano K. Kaneko R. Izawa T. Kawaguchi M. Kitsukawa T., 2012 Single-neuron diversity generated by protocadherin-β cluster in mouse central and peripheral nervous systems. Front. Mol. Neurosci. 5(August): 1–13. 22969705 10.3389/fnmol.2012.00090 PMC3431597

[bib16] Huddleston J. Ranade S. Malig M. Antonacci F. Chaisson M., 2014 Reconstructing complex regions of genomes using long-read sequencing technology. Genome Res. 24(4): 688–696. 24418700 10.1101/gr.168450.113 PMC3975067

[bib39] Hulpiau P. van Roy F., 2009 Molecular evolution of the cadherin superfamily. Int. J. Biochem. Cell. Biol. 41(2): 349–369. 18848899 10.1016/j.biocel.2008.09.027

[bib40] Jiang X.-J. Li S. Ravi V. Venkatesh B. Yu W. P. 2009 Identification and comparative analysis of the protocadherin cluster in a reptile, the green anole lizard. PloS One, 4(10): e7614. 19898614 10.1371/journal.pone.0007614 PMC2764143

[bib17] Kaneko R. Kato H. Kawamura Y. Esumi S. Hirayama T., 2006 Allelic gene regulation of Pcdh-alpha and Pcdh-gamma clusters involving both monoallelic and biallelic expression in single Purkinje cells. J. Biol. Chem. 281(41): 30551–30560. 16893882 10.1074/jbc.M605677200

[bib18] Keane T. M. Creevey C. J. Pentony M. M. Naughton T. J. McInerney J. O. 2006 Assessment of methods for amino acid matrix selection and their use on empirical data shows that ad hoc assumptions for choice of matrix are not justified. BMC Evol. Biol. 6: 29. 16563161 10.1186/1471-2148-6-29 PMC1435933

[bib19] Kehayova P. Monahan K. Chen W. Maniatis T. 2011 Regulatory elements required for the activation and repression of the protocadherin-alpha gene cluster. Proc. Natl. Acad. Sci. USA 108(41): 17195–17200. 21949399 10.1073/pnas.1114357108 PMC3193253

[bib42] Kise Y. Schmucker D., 2013 Role of self-avoidance in neuronal wiring. Curr. Opin. in Neurobiol. 23(6): 983–989. 10.1016/j.conb.2013.09.011 24161734

[bib43] Kohmura N. Senzaki K. Hamada S. Kai N. Yasuda R., 1998 Diversity revealed by a novel family of cadherins expressed in neurons at a synaptic complex. Neuron 20(6): 1137–1151. 9655502 10.1016/s0896-6273(00)80495-x

[bib20] Li H. Handsaker B. Wysoker A. Fennell T. Ruan J., 2009 The Sequence Alignment/Map format and SAMtools. Bioinformatics 25(16): 2078–2079. 19505943 10.1093/bioinformatics/btp352 PMC2723002

[bib45] Nicoludis J. M. Lau S. Y. Scharfe C. P. Marks D. S. Weihofen W. A., 2015 Structure and Sequence Analyses of Clustered Protocadherins Reveal Antiparallel Interactions that Article Structure and Sequence Analyses of Clustered Protocadherins Reveal Antiparallel Interactions that Mediate Homophilic Specificity. Structure 23(11): 2087–2098. 26481813 10.1016/j.str.2015.09.005 PMC4635037

[bib21] Noonan J. P. Grimwood J. Danke J. Schmutz J. Dickson M., 2004 a Coelacanth genome sequence reveals the evolutionary history of vertebrate genes. Genome Res. 14(12): 2397–2405. 15545497 10.1101/gr.2972804 PMC534663

[bib22] Noonan J. P. Grimwood J. Schmutz J. Dickson M. Myers R. M. 2004 b Gene conversion and the evolution of protocadherin gene cluster diversity. Genome Res. 14(3): 354–366. 14993203 10.1101/gr.2133704 PMC353213

[bib23] Paranjpe S. S. Jacobi U. G. van Heeringen S. J. Veenstra G. J. 2013 A genome-wide survey of maternal and embryonic transcripts during *Xenopus tropicalis* development. BMC Genomics 14: 762. 24195446 10.1186/1471-2164-14-762 PMC3907017

[bib24] Ribich S. Tasic B. Maniatis T., 2006 Identification of long-range regulatory elements in the protocadherin-alpha gene cluster. Proc. Natl. Acad. Sci. USA 103(52): 19719–19724. 17172445 10.1073/pnas.0609445104 PMC1750919

[bib48] Rubinstein R. Thu C. A. Goodman K. M. Wolcott H. N. Bahna F., 2015 Molecular logic of neuronal self-recognition through protocadherin domain interactions. Cell 163(3): 629–642. 26478182 10.1016/j.cell.2015.09.026 PMC4624033

[bib37] Sano K. Tanihara H. Heimark R. L. Obata S. Davidson M., 1993 Protocadherins: a large family of cadherin-related molecules in central nervous system. EMBO J. 12(6): 2249–2256. 8508762 10.1002/j.1460-2075.1993.tb05878.x PMC413453

[bib25] Schreiner D. Weiner J. A. 2010 Combinatorial homophilic interaction between gamma-protocadherin multimers greatly expands the molecular diversity of cell adhesion. Proc. Natl. Acad. Sci. USA 107(33): 14893–14898. 20679223 10.1073/pnas.1004526107 PMC2930437

[bib26] Sievers F. Wilm A. Dineen D. Gibson T. J. Karplus K., 2011 Fast, scalable generation of high-quality protein multiple sequence alignments using clustal omega. Mol. Syst. Biol. 7(539): . 10.1038/msb.2011.75 PMC3261699 21988835

[bib27] Tan M. H. Au K. F. Yablonovitch A. L. Wills A. E. Chuang J., 2013 RNA sequencing reveals a diverse and dynamic repertoire of the *X. tropicalis* transcriptome over development. Genome Res. 23(1): 201–216. 22960373 10.1101/gr.141424.112 PMC3530680

[bib28] Tan Y.-P. Li S. Jiang X. J. Loh W. Foo Y. K., 2010 Regulation of protocadherin gene expression by multiple neuron-restrictive silencer elements scattered in the gene cluster. Nucleic Acids Res. 38(15): 4985–4997. 20385576 10.1093/nar/gkq246 PMC2926608

[bib41] Thu C. A. Chen W. V. Rubinstein R. Chevee M. Wolcott H. N., 2014 Single-Cell Identity Generated by Combinatorial Homophilic Interactions between α, β, and γ Protocadherins. Cell 158(5): 1045–1059. 25171406 10.1016/j.cell.2014.07.012 PMC4183217

[bib29] Trapnell C. Pachter L. Salzberg S. L., 2009 TopHat: discovering splice junctions with RNA-seq. Bioinformatics 25(9): 1105–1111. 19289445 10.1093/bioinformatics/btp120 PMC2672628

[bib30] Wernersson R. Pedersen A. G., 2003 RevTrans: multiple alignment of coding DNA from aligned amino acid sequences. Nucleic Acids Res. 31(13): 3537–3539. 12824361 10.1093/nar/gkg609 PMC169015

[bib31] Wu Q., 2005 Comparative genomics and diversifying selection of the clustered vertebrate protocadherin genes. Genetics 169(4): 2179–2188. 15744052 10.1534/genetics.104.037606 PMC1449604

[bib32] Wu Q. Zhang T. Cheng J. F. Kim Y. Grimwood J., 2001 Comparative DNA sequence analysis of mouse and human protocadherin gene clusters. Genome Res. 11(3): 389–404. 11230163 10.1101/gr.167301 PMC311048

[bib44] Wu Q. Maniatis T., 1999 A striking organization of a large family of human neural cadherin-like cell adhesion genes. Cell 97(6): 779–790. 10380929 10.1016/s0092-8674(00)80789-8

[bib33] Yang Z., 2007 PAML 4: Phylogenetic Analysis by Maximum Likelihood. Mol. Biol. Evol. 24(8): 1586–1591. 17483113 10.1093/molbev/msm088

[bib34] Yokota S. Hirayama T. Hirano K. Kaneko R. Toyoda S., 2011 Identification of the cluster control region for the protocadherin-beta genes located beyond the protocadherin-gamma cluster. J. Biol. Chem. 286(36): 31885–31895. 21771796 10.1074/jbc.M111.245605 PMC3173131

[bib46] Yu W.-P. Yew K. Rajasegaran V. Venkatesh B., 2007 Sequencing and comparative analysis of fugu protocadherin clusters reveal diversity of protocadherin genes among teleosts. BMC Evol. Biol., 7: 49. 17394664 10.1186/1471-2148-7-49 PMC1852091

[bib35] Yu W.-P. Rajasegaran V. Yew K. Loh W. L. Tay B. H., 2008 Elephant shark sequence reveals unique insights into the evolutionary history of vertebrate genes: a comparative analysis of the protocadherin cluster. Proc. Natl. Acad. Sci. USA 105(10): 3819–3824. 18319338 10.1073/pnas.0800398105 PMC2268768

[bib47] Zipursky S. L. Sanes J. R., 2010 Chemoaffinity revisited: Dscams, protocadherins, and neural circuit assembly. Cell 143(3): 343–353. 21029858 10.1016/j.cell.2010.10.009

