# Supplementary material for: Characterization of a Single Genomic Locus Encoding the Clustered Protocadherin Receptor Diversity in Xenopus tropicalis
Source: G3 (Bethesda). 2016 Jun 3;6(8):2309–18. doi: 10.1534/g3.116.027995 (PMC4978886; doi:10.1534/g3.116.027995)
Supplement: Supplemental Material [file supp_6_8_2309__index.html]

Characterization of a Single Genomic Locus Encoding the Clustered Protocadherin Receptor Diversity in Xenopus tropicalis — Supplemental Material 

# Characterization of a Single Genomic Locus Encoding the Clustered Protocadherin Receptor Diversity in *Xenopus tropicalis*

## Supplemental Material for Etlioglu *et al.*, 2016

**Files in this Data Supplement:**

- Figure S1 - Heat map showing the pairwise sequence identities of the EC2-EC3 of mouse and X.tropicalis cPcdh isoforms at the amino acid level. (.pdf, 329 KB)
- Figure S2 - Phylogenetic relationships of mouse and *Xenopus tropicalis* cPcdhs. (.pdf, 400 KB)
- Figure S3 - Multiple sequence alignment of representative mouse and *Xenopus tropicalis* alpha-protocadherins. (.pdf, 329 KB)
- Figure S4 - Multiple sequence alignment of a representative mouse gamma-protocadherin and a *Xenopus tropicalis* gamma1 clustered protocadherin. (.pdf, 381 KB)
- Figure S5 - Multiple sequence alignment of a representative mouse gamma-protocadherin and a *Xenopus tropicalis* gamma2 clustered protocadherin. (.pdf, 366 KB)
- File S1 - Amino Acid Sequences of Xenopus tropicalis clustered Protocadherins. (.txt, 89 KB)
