# Supplementary material for: Characterization of a Single Genomic Locus Encoding the Clustered Protocadherin Receptor Diversity in Xenopus tropicalis
Source: G3 (Bethesda). 2016 Jun 3;6(8):2309–18. doi: 10.1534/g3.116.027995 (PMC4978886; doi:10.1534/g3.116.027995)
Supplement: Supplemental Material [file supp_g3.116.027995_FigureS2.pdf]

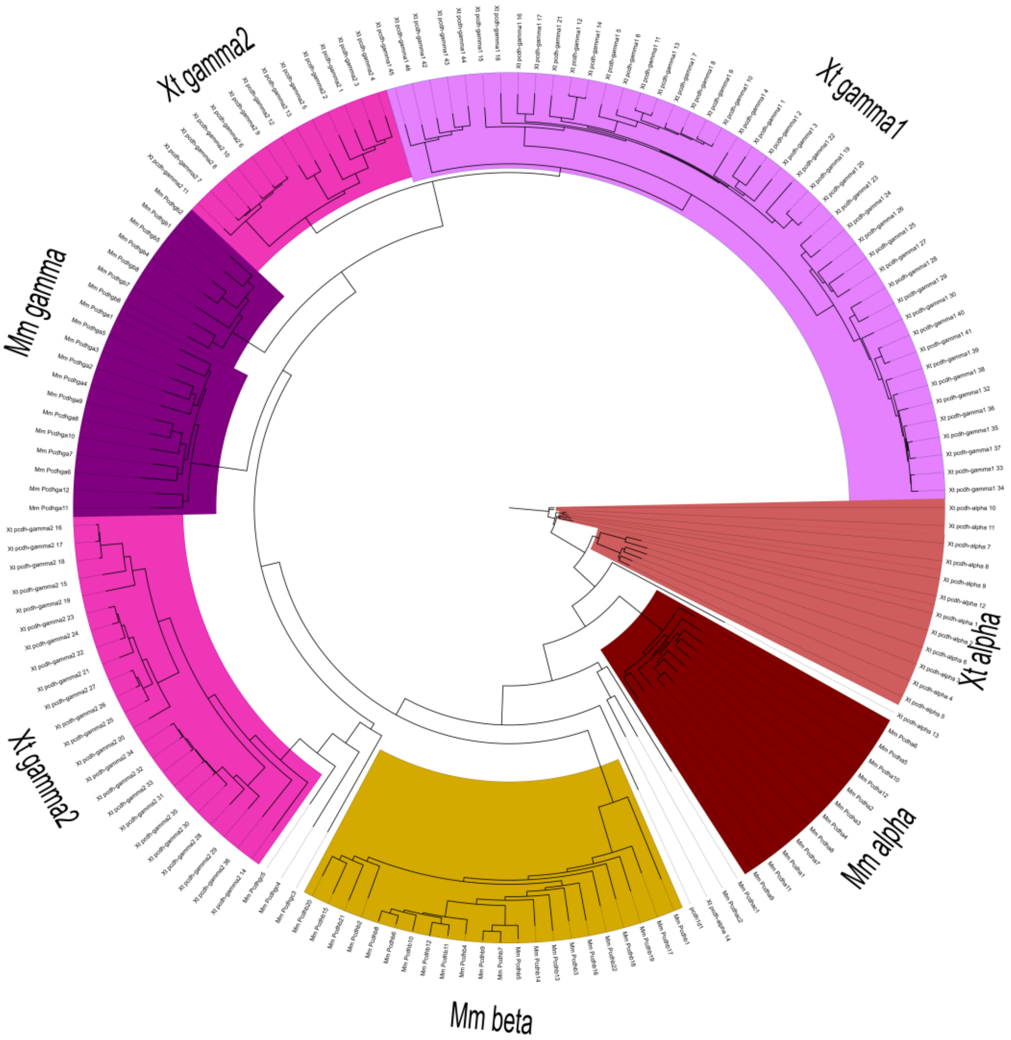

Supplementary Figure 2

Figure S2: Phylogenetic relationships of mouse and *Xenopus tropicalis* cPcdhs. The tree was generated with Maximum Likelihood using the multiple sequence alignments of full-length mouse and *Xenopus tropicalis* cPcdh isoforms.
